# Non-Egalitarian Allocations among Preschool Peers in a Face-to-Face Bargaining Task

**DOI:** 10.1371/journal.pone.0120494

**Published:** 2015-03-18

**Authors:** Alicia P. Melis, Anja Floedl, Michael Tomasello

**Affiliations:** 1 Warwick Business School (Behavioural Science group), University of Warwick, Coventry, United Kingdom; 2 Max-Planck Institute for evolutionary Anthropology, Department of developmental and comparative psychology, Leipzig, Germany; 3 Klinik für psychische Erkrankungen, Saale-Unstrut Klinikum Naumburg, Naumburg/Saale, Germany; CNR, ITALY

## Abstract

In face-to-face bargaining tasks human adults almost always agree on an equal split of resources. This is due to mutually recognized fairness and equality norms. Early developmental studies on sharing and equality norms found that egalitarian allocations of resources are not common before children are 5 or 6 years old. However, recent studies have shown that in some face-to face collaborative situations, or when recipients express their desires, children at much younger ages choose equal allocations. We investigated the ability of 3.5 and 5-year-olds to negotiate face-to-face, whether to collaborate to obtain an equal or an unequal distribution of rewards. We hypothesized that the face-to-face interaction and interdependency between partners would facilitate egalitarian outcomes at both ages. In the first experiment we found that 5-year-olds were more egalitarian than 3.5-year-olds, but neither of the age classes shared equally. In the second experiment, in which we increased the magnitude of the inequality, we found that children at both ages mostly agreed on the unequal distribution. These results show that communication and face-to-face interactions are not sufficient to guarantee equal allocations at 3–5 years of age. These results add to previous findings suggesting that in the context of non-collaboratively produced resources it is only after 5 years of age that children use equality norms to allocate resources.

## Introduction

Early research on the development of fairness found that it is only from 5–6 years onwards that children exhibit inequality aversion. In the majority of these early studies, subjects were interviewed and asked how they would allocate rewards between two hypothetical partners [1–3]. In some studies children were participants and recipients of the rewards but the partner was still a fictitious one, in order to rule out the possibility that the children’s behaviour might be motivated by the expectation of future favours by the partner [4, 5]. In all these hypothetical scenarios using verbal measures, the youngest children generally behaved selfishly and it was only from around 5 or 6 years of age that they tended to divide resources equally (equitable allocations emerged even later from 7–8 years onwards).

More recently, economic games that investigate altruism and equality have found that, when subjects are involved and make real decisions, winning more or less rewards depending on their choices, children at younger ages (4–5 years of age), sometimes choose to altruistically share resources with others, and that they even share low-value resources in an egalitarian way [6,7]. Similarly, in mini-dictator games in which children are given the choice between two predetermined options resulting in equal or unequal allocations (e.g. 1 reward for the subject and 1 for the recipient or 1 reward only for the subject), there are some occasions in which children younger than 7 choose the equal option although choosing equality in these situations is generally associated with low or no costs for themselves [8, 9]. In the study by Brownell et al. [9], 24-month-olds chose the non-costly equal option (1–1 over 1–0), but only when the recipient communicated her desire. Moore [10] found that in the more costly condition in which children had to choose between 2 rewards for themselves now and 0 for the partner or 1 for each later, only in around 50% of the trials did children choose the equal option and this mainly occurred among friends (sharing less with strangers and familiar non-friends) [11]. All these studies show that when subjects make real choices with real consequences, equality does emerge before 5–6 years of age, although it still does not happen in the great majority of the trials (in approximately 67% of the trials in Thompson et al. [8] and/or is generally non-costly.

Fehr, Bernhard and Rockenbach [12] reported a similar pattern regarding non-costly equal choices in their study with 3- to 8-year-olds. In this study children were presented with an option between equal (1–1) and unequal allocations (prosocial trials: 1–0; envy trials: 1–2; sharing trials: 2–0). As in previous studies 3–4-year olds chose the equal option only when this was associated with no costs for themselves (prosocial trials) or the partners would have been better off than themselves (envy trials). Only 8-year-olds showed disadvantageous and advantageous inequality aversion and were willing to pay a cost to maintain equality, preferentially choosing the equal allocation in the sharing trials (although see [13] who found, across different cultures, that children younger than 6 years of age engaged in costly prosociality). Recently, Blake & McAuliffe [14] found that in a mini-ultimatum game conducted among unfamiliar peers of 4- to 8-year-olds, children exhibited aversion to disadvantageous inequality from 4 years of age (see also [15], and were willing to forego a candy to avoid an unequal and disadvantageous allocation. However, aversion to advantageous inequality did not emerge until 8 years of age, when children refused 4 candies to prevent their peer from receiving less than them. The results from all these studies suggests that although children are from 4 years of age on sensitive to disadvantageous payoffs, there is a strong selfish bias at these young ages, and it is only later in ontogeny when they are willing to pay a cost incorporating equality norms in their interactions with others.

### Interactive sharing contexts

In the studies reviewed above subjects make solo decisions on how to distribute the resources, but a few studies have also investigated the development of equality in interactive sharing situations. Birch & Billman [16] took a first step in this direction and distributed food items unequally between pairs of 3–5-year-olds; the target child received 10 food pieces whereas the partner was given only 1. On average children shared only 1.5 pieces and sharing was mainly elicited by the recipient. Thus, these results replicated previous findings suggesting that children below 6–8 years of age are not willing to pay a cost to guarantee equal allocations.

A more recent line of studies in which peers interact face-to-face and both individuals in a dyad can influence the final outcome has found that collaboration or joint acquisition of resources enhances equality. In these studies pairs of 3-year-olds shared equally (around 75% of the trials) when they had acquired the resources collaboratively by simultaneously pulling on a rope in order to move a baited board within reach. However, they did not share equally when they pulled independently of each other (parallel work condition), or did not pull at all, since the rewards were already reachable from the start of the trial (windfall situation) ([17, 18], see also [19] for similar results with a puppet partner who intentionally chose not to help). These studies show that 3-year-olds are sensitive to outcomes produced in collaborative interactions and that this positively influences their sharing behaviour towards equality in face-to-face interactions. It also suggests a different developmental trajectory for the allocation of collaboratively and non-collaboratively produced resources.

In the previous interactive studies although peers could talk to each other and influence the final allocation, decisions to share could also be made unilaterally by one of the children. However, little is still known about the development of equality in bargaining situations in which individuals must come to an agreement before any participant can obtain anything. This is a highly relevant and ecologically valid situation from a fairly young age, since often children cannot make unilateral decisions and depend on the partner to initiate and maintain collaboration in a way satisfactory for both (e.g. in play contexts).

Previous developmental studies looking at face-to-face bargaining have generally investigated children older than six [20–22]. For example, in a study by Morgan & Sawyer [21], 10–12 years-old boys had to choose one of several possible predetermined money allocations (ranging from unequal to equal). Both friends and non-friends showed a strong preference for equality. However, friends were also willing to accept less favourable allocations. Generally, the results from these studies suggest a strong preference for equality allocations even overcoming equity concerns, maybe influenced by the loss of anonymity, and the effect of peer pressure, friendship and reputation concerns [22].

### The current study

In the present study we were interested in the level of equality exhibited by pairs of 3.5 and 5-year-olds when they are given the choice between an equal and an unequal distribution of rewards. How do children solve a conflict of interests in which one child’s best possible outcome is associated with a worse deal for the partner? From which age can they overcome self-serving biases and agree on mutually acceptable and fair outcomes? It has recently been suggested [23] that young children’s low responses to inequality and others’ material desires could be due to the difficulty in recognizing others’ needs and not to a lack of motivation to share, since when desires for material goods are made explicit children more readily behave prosocially [9,24]. If this is the case, a bargaining situation in which peers can verbally and physically express what they want should facilitate the emergence of equality.

We presented dyads of 3.5- and 5-year-old children with two baited boards, one holding 2 rewards for each child and the other holding 3 (or 6 in Experiment 2) rewards for one child and 1 reward for the other child. Children were required to pull simultaneously to move the board within reach and were instructed to choose only one of the boards. We chose a collaboration task to give both children in the dyad bargaining power and create interdependency between partners. However, it is important to emphasize that the negotiation process took place prior to collaboration. The study resembles previous studies, in which children could choose between two predetermined options with equal or unequal resource distribution [10–12], but with the difference that it was not a dictator-type game but instead an open bargaining game in which children could negotiate face-to-face about which option to work for (resembling also an ultimatum game, where partners can refuse the proposed deal).

We chose 3.5-year-olds since the recent collaboration studies mentioned above have shown that 3-year-olds can occasionally share equally. In addition, we tested 5-year-olds since many studies have shown that with increasing age children become increasingly prosocial and more egalitarian when distributing resources [7, 11, 25, 26]. Furthermore, studies on delay of gratification and future-oriented behaviour have shown a clear developmental change at this age, in the sense that children become more capable of waiting for larger rewards for themselves and others [8, 27]. This capacity could also help children in the present task, since children could potentially reach the highest levels of efficiency by agreeing on taking turns obtaining the unequal but advantageous deal.

Our hypothesis was that at 5 year of age, if not earlier, children would be able to agree on an equal and egalitarian outcome. Since previous research has shown that from 3 years of age children react negatively to disadvantageous inequality [14,15] and communication and collaboration increases levels of prosociality [9,18], we hypothesize that in the present task the children with the most unfavourable deal would react negatively and the interaction face-to-face would upset their partners. Due to peer pressure and children’s readiness to pay a cost to avoid disadvantageous inequality, dyads at both ages or the latest at 5 years of age, would end up agreeing on the equal tray.

Alternatively, based on previous studies showing that children younger than 8 are rarely willing to pay a cost to reduce advantageous inequality, and in the present task choosing the equal option is costly (children would lose 1 and 4 rewards in Experiments 1 and 2 respectively) one could also hypothesize that they would struggle to agree on the equal option.

Since gender, familiarity and friendship between partners have been found to play a role in how children’s make decisions about rewards’ allocations, we also included these variables in the analysis. Gender differences have not always been found but when they emerge it is normally girls that behave more prosocially [7, 28, although see 1]. Regarding friendship, as mentioned above, friendship has been found to be associated with more equality in bargaining situations [21] and more prosociality in sharing situations [10,16].

## Experiment 1: Inequality game (1–3 vs. 2–2)

### Methods

#### Participants

We tested 48 preschoolers in same-sex dyads (with equal numbers of boys and girls). Half of the dyads (N = 12) were 3.5-year-olds (*M* = 3.9 years, range = 3.5–3.10 years) and the other half (N = 12) 5-year-olds (*M* = 5.4 years, range = 5.0–5.5 years). An additional nine dyads with 3.5-year-olds were tested, but these were not included in the final sample due to equipment failure (one female dyad) and not passing the pre-test within 9 trials (five female dyads; three male dyads).

Children were recruited from the database of the Department of Developmental and Comparative Psychology at the Max Planck Institute for Evolutionary Anthropology in Leipzig, Germany. Most children came from middle- to upper-class families. The institutional ethics committee at the MPI for Evolutionary Anthropology approved the study. All parents provided written informed consent for their children’s participation in the study.They were tested at local day-care centres and paired either with a familiar partner (“familiar”) with whom they spent a lot of time playing in the same class during day care (as reported by the teachers) or with an unfamiliar partner (“unfamiliar”) they did not know, but who also attended the same day-care centre. Unfamiliar partners were thus not disliked peers but strangers or acquaintances at the most, whereas familiar partners were friends

#### Apparatus and set up

**Fig 1:**
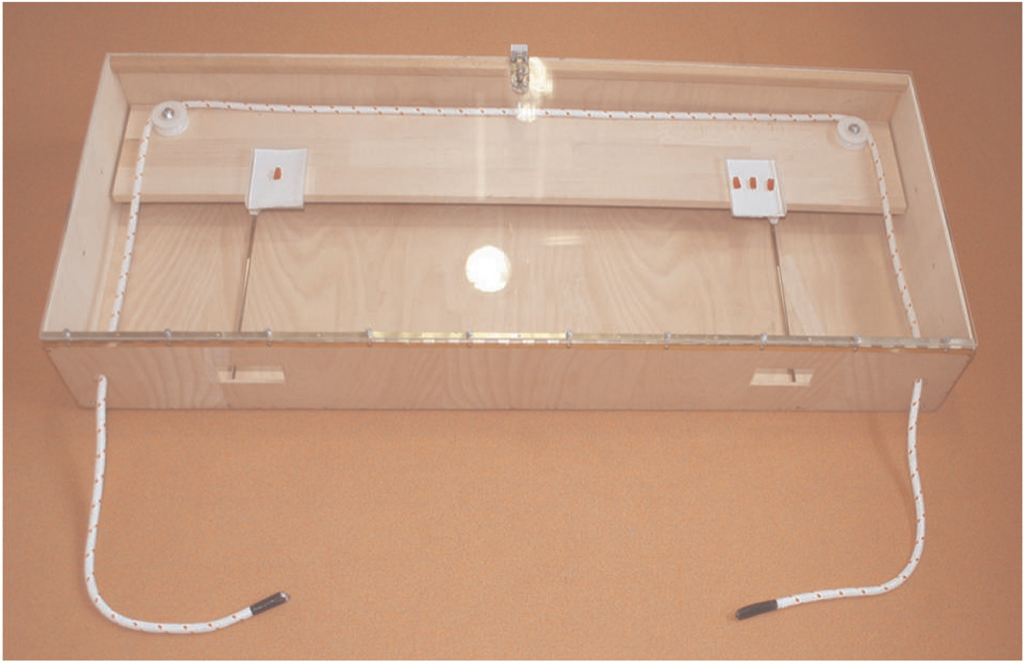
Cooperation apparatus showing the unequal split of gummy bears of Experiment 1.

The cooperation apparatus was adapted from studies with chimpanzees [29, 30] but had already been used with children as well [18]. We used two identical apparatuses. Each apparatus consisted of a wooden box with a transparent cover (115×45×15 cm) (Fig. 1). The rewards (gummy bears) were placed on little plates (8×10 cm) fixed onto a board inside the box. At the beginning of each trial, the board was located at the rear end of the box. In order to pull the board towards the front of the box, children had to pull simultaneously on a rope (295 cm long and 1.3 cm in diameter), which was looped around two wheels that were fixed on the board. Each end of the rope (50 cm) was sticking out of the box through holes at the front of the apparatus, and each child had to hold one end of the rope and pull simultaneously with the other to move the board towards the front of the box. If only one child pulled her end of the rope, the rope came out of the wheels, in turn becoming disconnected from the board. Once children pulled simultaneously the board moved towards the front part of the box, and children could then access the gummy bears by reaching through the two windows (8×4 cm each) right in front of them. Theoretically, it was possible for one child alone to pull both ends of the rope simultaneously. We therefore placed the ends of the rope along the sides of the apparatus, so that it was not too obvious for a child to think about pulling both ends of the rope. In addition, we implemented the rule that children were not allowed to pull both ends of the rope on their own.

The two apparatuses were placed 40–70 cm apart in a quiet room at the day-care centre, in such a way that both apparatuses were equidistant from the door through which the children entered.

#### Procedure and design

*Warm-up phase*: This phase consisted of first playing with each child separately in their playgroup. Then, children were asked if they wanted to participate in a game together with a partner. Children were then paired up with the partner (half of the children were paired with a familiar and the other half with an unfamiliar partner) and went with the experimenters (E1 and E2) into a room, which was next to the testing room. To make the children feel more comfortable with their new surroundings—and, in half of the cases, with a new partner—E1 and E2 looked at a picture book together with the children. Afterwards, E1 showed the children a bag of gummy bears and told them that they could play a game in the room next door, in which they could win lots of gummy bears.

*Demonstration phase*: The two experimenters sat in front of the apparatus and demonstrated how to move the board within reach by holding onto both ends of the rope and pulling them simultaneously. Meanwhile the children were sitting or standing between E1 and E2 and just watching the demonstration. This demonstration was conducted with both apparatuses. During the demonstration the plates in both apparatuses were filled with the same amount of gummy bears (3 gummy bears per plate). After the demonstration children went out of the testing room with E1.

*Introduction phase*: It was important ensuring that children understood the game and paid attention to the contents of the 2 apparatuses before making a choice. Therefore, to acquaint children with the procedure, we introduced them to all important information little by little (E1 becoming increasingly passive in the introduction and staying outside the testing room during the pretest phase). The children waited with E1 outside the testing room while E2 baited the apparatuses. After E2 returned, the children entered the testing room together with E1. The introduction phase was split into two parts. In the first 2 trials, E1 helped the children with the order of events in the game by (1) checking with them both apparatuses and the amount of gummy bears in each apparatus and on each plate; (2) encouraging children to decide together which apparatus they wanted; and (3) after reaching a joint decision and the children pulling on the rope together, telling the children that each child would get the gummy bears of her/his plate, and that they shouldn’t split differently the gummy bears obtained (“Each keeps her own gummy bears”). It was important to tell the children that they should not share the gummy bears obtained, since we were interested in testing whether or not they would come up with an egalitarian solution before collaboration (Hamann et al. [17] have already shown that they restore equality after collaboration). The plates in the 2 apparatuses were baited in two possible ways: 0–0 vs. 3–3 and 1–1 vs. 3–3. In the second part of the introduction (2–4 trials, depending on how well children understood the rules), E1 gave instructions to the children regarding how to behave and play the game. E1 said: *“I will repeat again how this game works*. *First*, *you look at one of the boards and then at the other board*. *Then you have to decide which box you both want*, *but everybody keeps their own gummy bears*. *Now it’s your turn*”. E1 remained in the background and only intervened if children forgot to look inside both apparatuses before deciding to pull one. The plates in the 2 apparatuses were baited in two possible ways: 0–0 vs. 2–2 and 1–1 vs. 3–3. In this phase each child was given a colour-marked but transparent tube (“gummy bear tower”) and told to place their gummy bears into the tube to take with them afterwards.

*Pre-test phase*: This phase was designed to ensure that children understood and adhered to the game rules and were motivated to obtain the largest possible amount of gummy bears. Children waited outside the testing room while E2 baited the apparatuses. Both plates in each apparatus were baited with the same amount of gummy bears. However, one apparatus had a larger amount of gummy bears than the other apparatus. There were three types of trials depending on the number of gummy bears per plate and apparatus: 1–1 vs. 2–2; 1–1 vs. 3–3; and 2–2 vs. 3–3. The side of the apparatus containing the largest amount was counterbalanced across trials. Pairs were required to succeed in pulling the apparatus with the largest amount of gummy bears in three consecutive trials. All pairs participated in a minimum of three and a maximum of nine trials. If after nine trials subjects were still inconsistently choosing different apparatuses (or consistently choosing the apparatus with less gummy bears) they were excluded from the test phase. On average 3.5-year-old dyads passed this pre-test after 5.3 trials and 5-year-old dyads after 3.9 trials (eight 3.5-year-old dyads were excluded as mentioned above).

*Test phase*: In this phase children were faced with the choice between an equal apparatus (2 gummy bears per plate) and an unequal apparatus (1 on one plate and 3 gummy bears on the other plate). The position of the apparatus (left or right) containing the unequal split was counterbalanced across trials and within dyads. The position of the plate (left or right) in the unequal apparatus holding the larger amount was also counterbalanced across trials and dyads. A trial started when children entered the testing room and ended either if they failed to retrieve the gummy bears (i.e. pulling out the rope or failing to succeed for 120 seconds) or after they had succeeded in retrieving the gummy bears. Each dyad participated in four test trials.

#### Coding and data analyses

A number of behavioural measures were coded within every trial. A “no agreement” occurred if children could not come to an agreement within two minutes, or if at least one child tried to pull the rope alone and it became unthreaded from the wheels, thus making the rope ineffective, or if the children argued so harshly that E1 had to intervene and stop the test. For “agreements” we discriminated between successful and unsuccessful agreements. Successful agreements were those cases in which children agreed on pulling one apparatus and actually succeeded in obtaining the gummy bears. Unsuccessful agreements also included those cases in which children had obviously agreed (verbally or non-verbally) to pull one apparatus but were not successful in getting the gummy bears because of coordination problems with the rope.

To investigate the distribution of gummy bears within the dyads, we coded the amount of gummy bears each child obtained (successful agreements) per trial. Furthermore, we coded which apparatus (equal or unequal distribution of gummy bears) the children chose and obtained. Additionally, we coded the protest behaviour between children in the negotiating phase of each trial. The coding phase started when children entered the testing room and lasted until the children started pulling the rope together. We observed and categorized as protest any of the following behaviours: verbal protests (e.g. “But I want three gummy bears!”, “No, that’s mine!”), gestural protests (e.g. head shaking, changing apparatus) and physical protest (e.g. pushing away the child from the favoured side of the apparatus). We only considered protests against a small amount in the unequal tray or the equal tray and did not distinguish whether one dyad used several types of protests within a single trial (trials were categorized as trials with protest or no protest/immediate acceptance). We coded whether or not the final decision of which tray dyads pulled was the result of any kind of protest or negotiation (“change of tray”). All coding was done from videotape by A.F. and A.P. M. A minimum of 20% of the trials were coded independently by a second person who was blind to the hypotheses tested (agreements, number of gummy bears per child and apparatus pulled: Cohen’s kappa = 1; protests: Cohen’s kappa = .808; change of tray: Cohen’s Kappa = .855).

*Effects of age*, *gender and familiarity of the partner on the equality of the rewards’ distribution*: The two response measures, one being the absolute difference of gummy bears within the dyads and the other the number of trials where the apparatus with an unequal distribution of gummy bears was chosen, were analysed using Generalized Linear Models (GLMs) separately for these two dependent variables in R (Version 2.10.1). For both GLMs the predictors were age group, gender and familiarity (see S1 Text for further details). For both analyses we always calculated percentages of choices of successful trials per dyad, since occasionally children did not succeed pulling any of the trays

*Equal*, *unequal or random allocation of rewards*: For the statistical analysis of the children’s decision to pull one or the other apparatus (equal vs. unequal) we used a Monte Carlo simulation (Manly, 1997). We ran two separate Monte Carlo simulations. For both analyses we simulated dyads making random decisions in favour of either of the two alternatives (equal or unequal apparatus), with the number of decisions equalling the number of successful trials per dyad, as revealed in the experiment. For the first analysis we determined for each dyad its absolute deviation from 50% decisions for one option (the random expectation) and then averaged these across dyads. The entire process was repeated 10,000 times. If this revealed significance, then the dyads had pure strategies. Having a pure strategy means that a certain dyad did not choose between the two trays randomly, but that instead chose significantly more often than expected by chance (50%) the equal or the unequal tray. However, the pure strategies were not necessarily the same across dyads. Therefore, we conducted a second analysis in which we determined the proportion of decisions for the unequal apparatus per dyad and then averaged these across dyads. The entire process was also repeated 10,000 times. If this reveals significance, pairs’ strategies were the same across dyads (most of the dyads choosing unequal or most of the dyads choosing equal). In both simulations we determined significance as the proportion of simulations revealing a score at least as far away from the expectation as the original data. The simulations were run in a script written for R [31] by R. Mundry.

To investigate whether, when children decided to pull the unequal apparatus, the overall distribution of gummy bears between the children was equal (over the four trials per condition), we compared (using an exact Wilcoxon signed-rank test) the absolute difference of gummy bears (per dyad) in the original data with an expected absolute difference of gummy bears (per dyad). In order to calculate the expected absolute difference (per dyad) we started by determining the number of decisions for the unequal apparatus (per dyad) and all possible distributions of the gummy bears for this number of sequences. For example, if the children decided twice to go for the unequal apparatus then there were the following possibilities: Either Child A or Child B got the big amount twice, or Child A got the big amount first and then Child B did, or vice versa. For all the possible distributions per dyad we determined the absolute difference of gummy bears received and then averaged them (revealing the expected value).In order to compare the overall distribution of rewards between children depending on how often they agreed to cooperate with the equal (vs. unequal) apparatus we plotted for each dyad (and for each age group) the proportion of gummy bears obtained by the “highest earner” child against the proportion of trials in which the dyad cooperated to obtain the unequal split. The highest earner was the child obtaining the largest amount of gummy bears within the dyad. We calculated two different potential theoretical distributions of rewards depending on whether or not, whenever dyads agreed to pull the unequal tray, there was alternation between individuals with regards to who obtained the larger amount (see S1 Text for further details).

Finally, we classified the patterns of each dyad as “Inequality”, “equality”, “reciprocal” and “mixed” depending on the overall outcome of their agreements. 1) Inequality pattern: One child obtained the big reward in the majority of the trials (at least 66% of trials, since children sometimes only succeeded in 3 trials); 2) Equality pattern: Children chose the equal split in the majority of the trials (at least 66% of trials); 3) Reciprocal pattern: Both children per dyad obtained the large reward as many times as the small reward; 4) Mixed pattern: Children chose different options so that no clear pattern could be defined.

### Results

**Fig 2:**
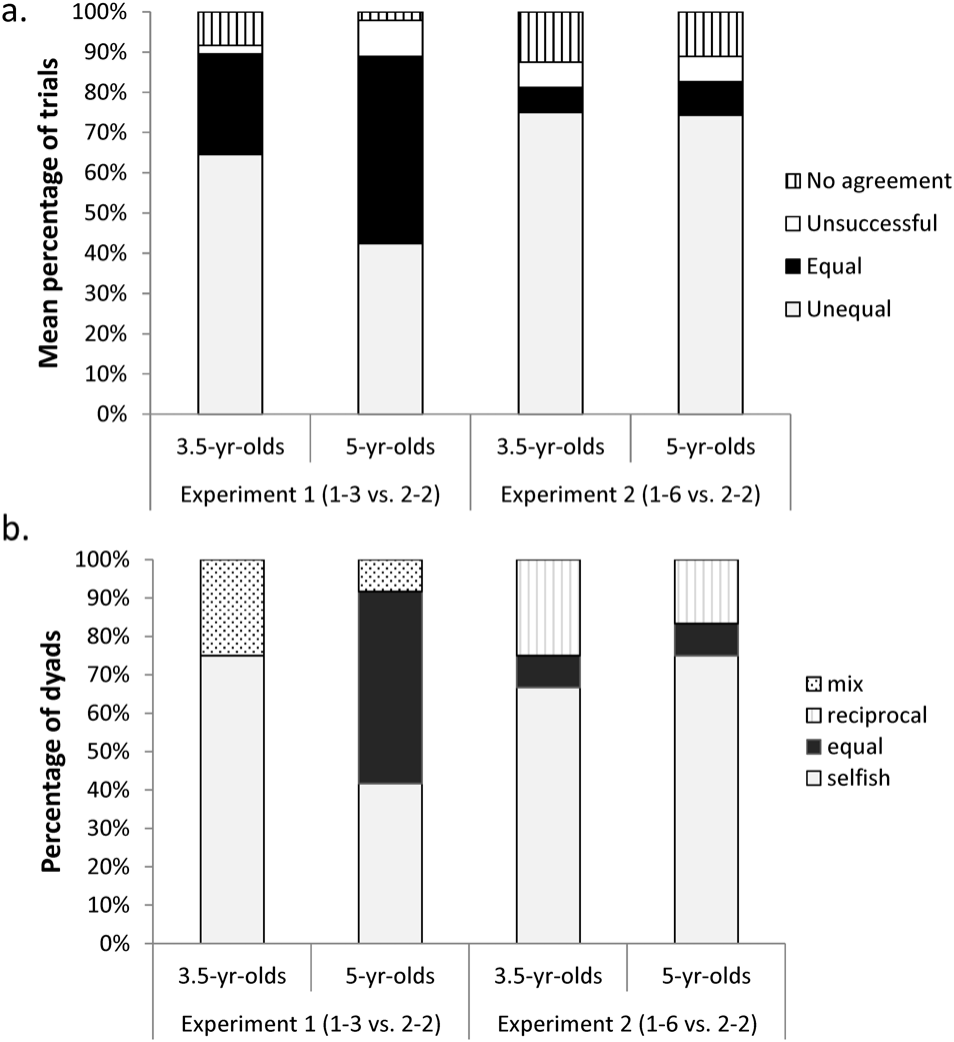
(a) Mean percentage of trials per age group and experiment (1 & 2) in which children did not come to an agreement, were unsuccessful due to coordination problems or agreed and collaborated obtaining the equal and unequal split. (b) Percentage of dyads per age group and experiment (1 & 2) that behaved “selfishly” (one child obtained the big reward in at least 66% of trials), “equally” (children chose the equal split in at least 66% of trials), “reciprocally” (both children obtained the large reward as many times as the small reward), in a “mixed” way (no clear pattern could be defined).

Both 3.5 and 5-year-olds agreed to cooperate by pulling one board in the majority of the trials (Mean percentage of trials per dyad: 3.5-yr-olds: mean ± SD = 92 ± 22% and 5-yr-olds: mean ± SD = 98 ± 7% of the trials, Fig. 2a). Overall, only 3 dyads were unable to come to an agreement (3.5-year-olds: one pair of girls in 1 trial and one pair of boys in 3 trials; 5-year-olds: one pair of girls in 1 trial). Children occasionally made coordination mistakes but they were successful in pulling the board within reach in the majority of the trials in which they came to an agreement (mean percentage of successful agreements: 3.5-year-olds (mean ± SD) = 98 ± 7%; 5-year-olds (mean ± SD) = 91 ± 13% of the trials, Fig. 2a). Overall, 3.5-year-olds agreed choosing the unequal split more often than the equal split (Mean percentage of trials per dyad choosing unequal and equal: 65± 23% and 25± 18% respectively; Wilcoxon signed-rank test: T = 45, N = 12, p = 0.007), whereas 5-year-olds chose both options (equal and unequal splits) equally often (unequal: 42± 39%, equal: 47± 36%; Wilcoxon signed-rank test: T = 31, N = 12, p = 0.86, see Fig. 2a).

#### Effects of age, gender and familiarity of the partner on the equality of the rewards’ distribution

In order to investigate how the factors age, gender and familiarity of the partner, influenced the final outcome, we conducted two independent GLMs for the two response measures: the absolute difference of gummy bears within the dyads and the number of trials choosing the unequal apparatus.

**Fig 3:**
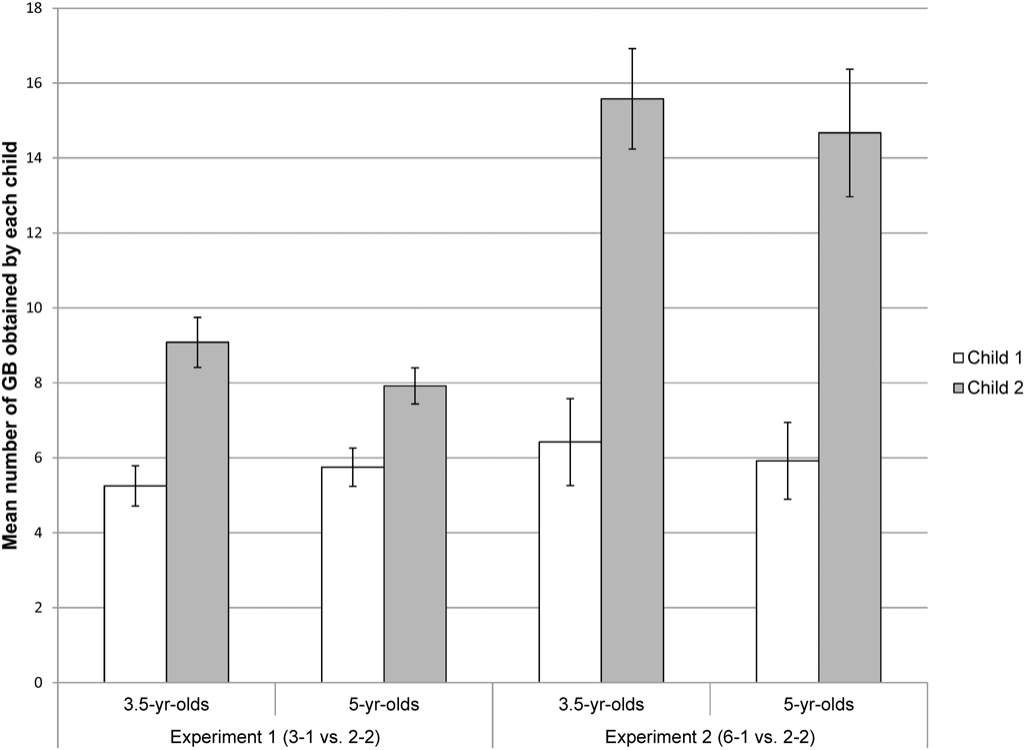
Mean absolute number of gummy bears (± SEM) obtained by the two children in a dyad in Experiments 1 and 2.

For the response variable “difference of gummy bears”, the full model including the variables gender, age and familiarity of the partner was highly significant compared to the null model (likelihood ratio test: X² = 8.73, df = 3, p = 0.03). More specifically, there was an age effect and a gender tendency on the overall difference of gummy bears within dyads. The difference of gummy bears within the 3.5-year-olds was greater than among the 5-year-olds (mean proportional difference of gummy bears among the 3.5-year-olds: mean ± SD = 28.4 ± 15% and 5-yr-olds: mean ± SD = 17 ± 17%, the maximal difference would be 50%), and it tended to be greater among the girls than the boys (see Tables A and B in S1 Text and Fig. 3). However, the variables gender, age and familiarity did not have any effect on the proportion of trials in which children chose the unequal apparatus (likelihood ratio test comparing the full versus the null model: X² = 4.83, df = 3, p = 0.18).

*Equal*, *unequal or random allocation of rewards*: Independently of whether or not the two age groups differed from each other, we were interested in testing whether the choices made at each age were rather “unfair”, “fair” or alternatively did not differ from chance outcomes. For this purpose we conducted two different analyses. First, we looked at whether children’s decisions to pull one or the other apparatus (equal vs. unequal) differed significantly from chance (50%). Second, we investigated whether children took turns getting the large reward when pulling the unequal tray, since this would be another solution leading to equality.

**Fig 4:**
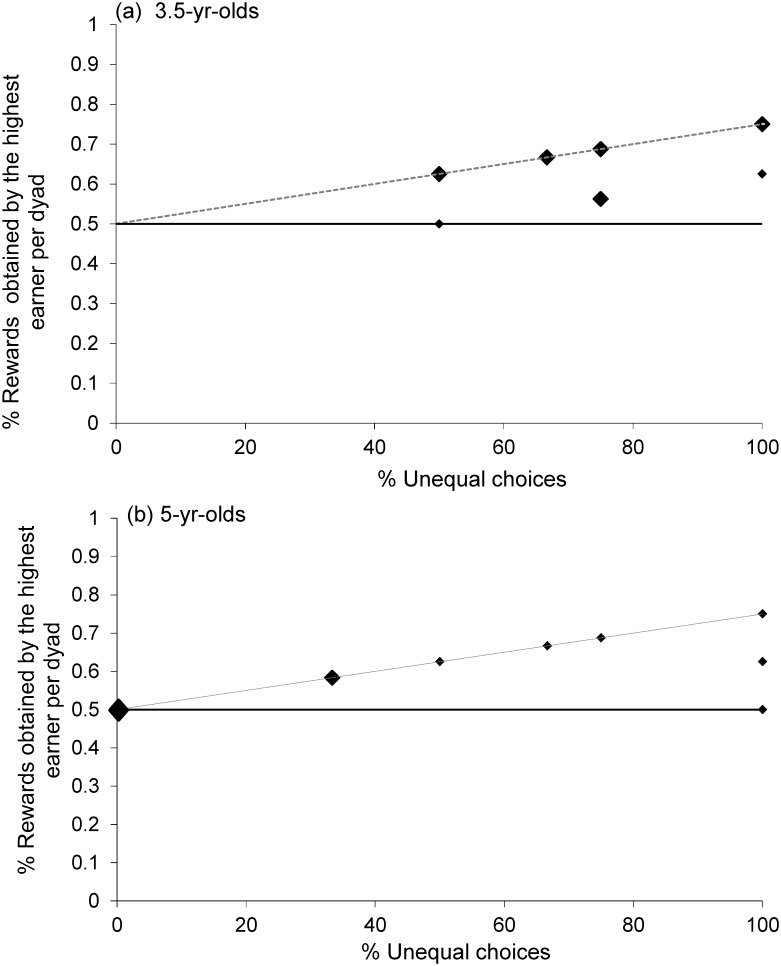
Proportion of rewards obtained by the “highest earner of each pair plotted against the proportion of trials in which the dyad pulled the unequal tray. The dashed lines (in a and b) describe the predicted outcome for the case in which the same child in a given pair always obtained the larger reward whenever they agreed to pull the unequal tray. The continuous lines describe the predicted outcome for the case in which individuals in a pair alternated who obtained the larger reward whenever they cooperated in order to obtain the unequal share (see SI). The bigger black dots represent two, three or four data points. N = 12 for each age group. There were nine dyads among the 3.5-year-olds choosing the unequal tray in 50%-75% of the trials but no dyad choosing the equal tray in more than 50% of the trials. Among the 5-year-olds, four dyads chose the equal tray in 100% of the trials and three dyads the unequal tray in 100% of the trials.

We ran two Monte Carlo simulations to look at whether children’s decisions to pull one or the other apparatus (equal vs. unequal) differed significantly from chance. In a first analysis we looked at whether dyads had pure strategies, that is, whether within individual dyads choices differed from 50% chance choosing equal or unequal. We found that 3.5-year-olds did not differ from chance (p = 0.45), whereas 5-year-olds did (p = 0.003). This means that within individual dyads 3.5-year-olds were not choosing one or the other tray significantly above chance levels, whereas 5-year-olds were. In order to see whether the strategies were the same across dyads, we ran a second Monte Carlo simulation. This revealed that the pure strategies of the 5-year-olds were not all in the same direction (p = 0.694), with some dyads agreeing on the equal and some dyads agreeing on the unequal apparatus. Fig. 4b shows how several dyads chose the unequal tray in 100% of the trials, and other dyads chose the equal tray in 100% of the trials. The 3.5-year-olds agreed more often than expected by chance on the unequal apparatus (p = 0.004) which means that there were not dyads on the equal side of the spectrum and dyads on the unequal side, but instead that all dyads tended to choose the unequal tray slightly more often (see Fig. 4a).

To investigate whether or not there was an overall fair distribution of rewards when children chose the unequal apparatus (if they chose the unequal apparatus 4 times, but took turns regarding who obtained the big amount, the final distribution would be completely equal), we compared the observed difference of gummy bears (per dyad) with an expected difference of gummy bears using a Wilcoxon signed-rank test (see methods to see how the expected value was calculated). We found that the final distribution of gummy bears among the 3.5-year-olds differed significantly from chance levels, in the sense that they were ‘more unequal’ (T = 56, N = 11, p = 0.044), whereas 5-year-olds did not (T = 15.5, N = 6, p = 0.31).

The results from the previous analyses are in line with the analyses of the first choices made. We looked at the first choices made (at trial 1) by each dyad and found that 3.5-year-olds chose the equal apparatus less often than expected by chance (only 2 out of 12 dyads chose the equal apparatus, binomial test: p = 0.038), whereas the choices of the 5-year-olds did not differ from chance (6 out of 12 chose the equal apparatus) (p = 1). Girls also chose the equal apparatus less often than expected by chance (only 2 out of 12 dyads chose the equal apparatus) (p = 0.038), whereas boys did not (6 out of 12 chose the equal apparatus, p = 1). Finally, familiar partners also chose the equal apparatus less often than predicted by chance (only 1 out of 12 dyads chose the equal apparatus, p = 0.006), whereas unfamiliar partners did not differ from chance levels (7 out of 12 dyads chose the equal apparatus, p = 0.77).

At a more descriptive level we were interested in the number of dyads exhibiting a pattern of behaviour that could be characterized as: “inequality”, “equality”, “reciprocal” and “mixed”. The characterization of the dyads revealed that nine dyads of 3.5-year-olds exhibited mostly inequality, whereas the other three behaved in a mixed way. Among the 5-year-olds, six out of the 12 dyads behaved in an equal way, five showed inequality and only one dyad behaved in a mixed way (Fig. 1b).

#### Description of the negotiation process

Finally, also at a descriptive level, we analysed two other measures (protests and changes in the decision of which tray to pull), that could shed light on how children negotiated and solved the conflict of interests. On average, 3.5-year-olds protested in 42% of trials (4 out of 12 dyads never protested at all) and 5-year-olds in 36% of trials (again, 4 dyads never protested, but 3 of them always agreed on pulling the equal tray). In most of the cases these protests were against the small amount (75% and 83% of protests among the 3.5-year-olds and 5-year-olds respectively). A small proportion of the protests were also against pulling the equal tray (25% and 17% of protests among the 3.5-year-olds and 5-year-olds respectively), which often occurred after the partner had obtained the large amount from the unequal apparatus in previous trials.

We analysed whether trials which ended on an unequal outcome differed from trials ending in an equal outcome with regard to two aspects: (1) which percentage of unequal/equal outcomes (per dyad) was preceded by some kind of protest vs. which percentage of trials was the result of both individuals immediately pulling or one child following the other one, without suggesting or commenting anything else (no protest), (2) which percentage of the unequal/equal outcomes was the result of children changing the tray which one of them initially suggested to pull. The levels of immediate acceptance of a tray (equal and unequal) and proportion of successful negotiation, leading to a change of trays, was not significantly different between the two age groups (Mann-Whitney U Test for all measures: NS). Children at both ages tended to immediately accept the equal and unequal tray at similar levels (in 50% of the trials). However, the proportion of equal outcomes due to dyads changing the tray that initially one of them intended to pull tended to be higher than the proportion of unequal outcomes due to a change of trays (Both ages together: Mean = 50% of trials with equal outcome vs. 21.13% of trials with unequal outcome; Wilcoxon exact Signed ranked Test: T^+^ = 30, n = 15, seven ties, p = 0.10). Note that in the 21% of the trials in which dyads accepted on pulling the unequal tray, one of the children managed to convince the partner to leave the equal tray in favour of the unequal tray (see S1 Fig).

### Discussion

Dyads at both ages came to an agreement in nearly all cases. However, differences appeared with regard to which apparatus they pulled and the final distribution of rewards. Several analyses confirmed that 3.5-year-olds exhibited more inequality than 5-year-olds. First, the difference of gummy bears within dyads was greater among the 3.5 than the 5-year-olds. Second, although individual 3.5-years-old dyads did not choose exclusively the unequal option, overall they agreed more often on the unequal apparatus, and when they did, the overall distribution of rewards between partners was unfair. In contrast, the pure strategies of the 5-year-olds varied, with some dyads agreeing more often on the equal and some dyads on the unequal apparatus. However, when 5-year-olds agreed on the unequal apparatus the distribution of rewards between partners did not differ from chance. This pattern of results could be observed from trial 1 in which the vast majority of 3.5-year-olds chose the unequal apparatus, whereas 5-year-olds chose the equal and unequal apparatus equally often. We didn’t find any clear age differences regarding how children behaved prior to pulling one or the other tray. When children protested against the unequal tray and there was no change of tray, they mostly said that they also wanted the large amount, and the discussion centred around the fact that both kids wanted the large amount, but not about the possibility of pulling the equal tray (and both getting a medium number of rewards). The age difference seems to have been due mainly to the higher number of dyads among the 5-year-olds that suggested right from the beginning to pull the equal tray, and not necessarily due to their better negotiation skills.

Although children could have chosen to pull the equal apparatus obtaining 2 gummy bears each, 3.5-year-olds agreed mostly on the unequal split and individuals obtaining the larger amount did not seem to care that their partner obtained a smaller amount of gummy bears even though he/she had contributed to obtaining the rewards. Five-year-olds exhibited more equality than the 3.5-year-olds, but they still did not behave in a purely egalitarian way (i.e. equality did not prevail).

These results are particularly striking considering that children often protested at and did not seem happy with the unequal and disadvantageous split. Furthermore, children who were going to receive the small amount could have refused to pull until the partner agreed to pull for the equal split. One possibility is that the magnitude of the inequality presented in this experiment was not high enough to make children care that much, and therefore they just tended to follow the partner independently of what (s)he wanted to obtain. It is possible that a higher level of inequality would motivate children, including 3.5 year-olds, to behave more fairly or motivate partners to refuse to pull in order to punish selfish partners and convince these to pull the equal apparatus. This was the rationale for the next experiment.

## Experiment 2: High-inequality game (1–6 vs. 2–2)

In this experiment we increased the magnitude of the inequality by placing six gummy bears instead of three on one of the plates of the unequal tray. We hypothesized that when presented with these 2 options, children at both ages would react more strongly to the inequality and would more likely agree on pulling the equal tray. Alternatively, another strategy to maximize the gains would be essentially to take turns regarding who obtains the large amount (6 pieces), thus over time benefiting both individuals in the pair (reciprocal strategy).

### Methods

#### Participants

As in the previous experiment we tested 48 preschoolers in dyads with a same-sex partner (with equal numbers of boys and girls). Half of the dyads (N = 12) were 3.5-year-olds (*M* = 3.9 years, range = 3.5–3.10 years) and the other half (N = 12) 5-year-olds (*M* = 5.3 years, range = 5.0–5.5 years). An additional five dyads were tested, but these were not included in the final sample due to experimenter errors (one dyad of 3.5-year-old females and one dyad of 5-year-old males) or because they did not pass the pre-test phase within 9 trials (three dyads of 3.5-year-old males; see below). Children who participated in this study had not participated in the previous one.

#### Apparatus and set up

The apparatuses and set up were identical to those described in Experiment 1.

#### Procedure and design

The procedure and design were exactly the same as in Experiment 1. On average 3.5-year-old dyads passed the pre-test after 4.25 trials and 5-year-old dyads after 4 trials (three 3.5-year-old dyads were excluded as mentioned above for not passing the pre-test).

The only difference was that in the test phase children were faced with the choice between an equal apparatus containing 2 gummy bears per plate and an unequal apparatus, containing 1 gummy bear on one plate and 6 gummy bears on the other plate.

#### Coding and analysis

The coding and analyses were performed in exactly the same way as in Experiment 1.

### Results

Both 3.5 and 5-year-olds agreed to cooperate by pulling one board in the majority of the trials (Mean percentage of trials per dyad, 3.5-yr-olds: mean ± SD = 88 ± 13% and 5-yr-olds: mean ± SD = 89 ± 27% of the trials, Fig. 2a). Overall, 9 dyads were unable to come to an agreement at least once (3.5-year-olds: Six dyads were unable to come to an agreement in 1 trial; 5-year-olds: Two dyads in 1 trial and 1 dyad in 3 trials). Children occasionally made coordination mistakes but they were successful in pulling the board within reach in the majority of the trials in which they came to an agreement (% successful agreements among 3.5-yr-olds: mean ± SD = 94 ± 15% and 5-yr-olds: mean ± SD = 94 ± 11% of the trials, Fig. 2a). Overall, both 3.5- and 5-year-olds agreed choosing the unequal split more often than the equal split (3.5-year-olds: unequal = 75± 21%, equal = 6± 16%; Wilcoxon signed-rank test: T = 77, N = 12, p = 0.002; 5-year-olds: unequal: 74± 32%, equal: 8± 22%; Wilcoxon signed-rank test: T = 72.5, N = 12, p = 0.008, see Fig. 2a).

#### Effects of age, gender and familiarity of the partner on the equality of the rewards’ distribution

In order to investigate the effects of age, gender and familiarity of the partner we conducted two independent GLMs for the two response measures: the absolute difference of gummy bears within the dyads and the number of trials where children chose the unequal apparatus.

Overall, the full model including the variables gender, age and familiarity was highly significant compared to the null model (likelihood ratio test: X² = 14.17, df = 3, p = 0.0026). More specifically, there were effects of gender and familiarity level, but no effects of age on the overall difference of gummy bears within dyads. The difference of gummy bears among the boys was greater than among the girls, and the difference was also greater among familiar partners than non-familiar partners (see Tables A and B in S1 Text and Fig. 3).

The variables gender, age and friendship did not have any effect on the proportion of trials in which children chose the unequal apparatus (likelihood ratio test comparing the full versus the null model: X² = 0.44, df = 3, p = 0.93).

#### Equal, unequal or random allocation of rewards

We conducted the same two analyses than in Experiment 1. First, we looked at whether children’s decisions to pull one or the other apparatus (equal vs. unequal) differed significantly from chance (50%). Second, we investigated whether children took turns getting the large reward when pulling the unequal tray, since this would be another solution leading to equality.

**Fig 5:**
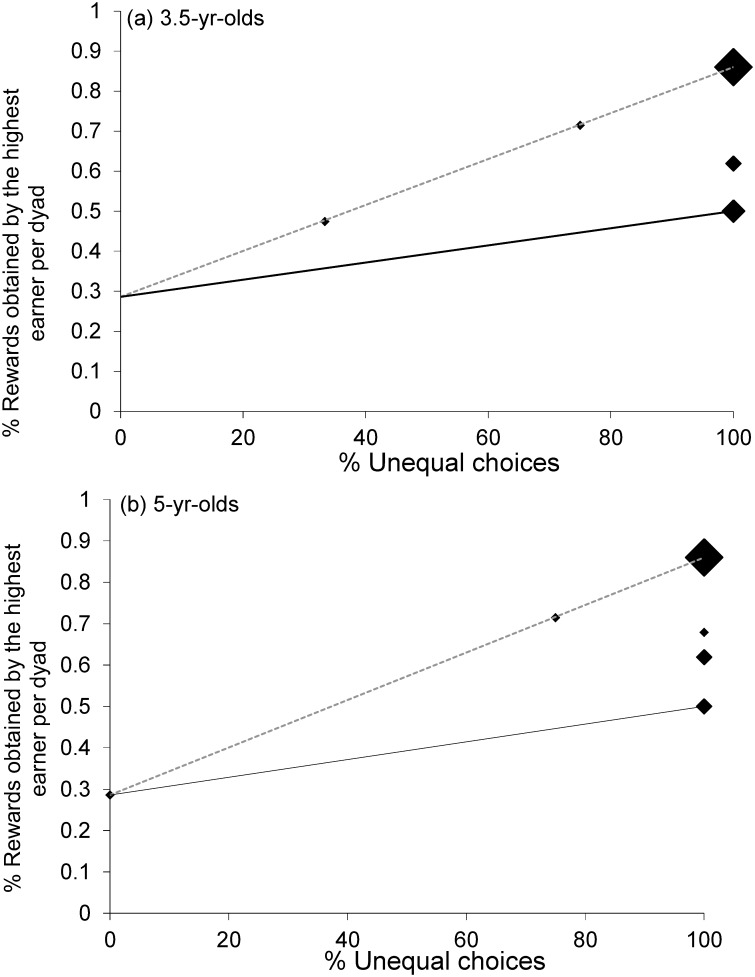
Proportion of rewards obtained by the “highest earner of each pair plotted against the proportion of trials in which the dyad pulled the unequal tray. The dashed lines describe the predicted outcome for the case in which the same child in a given pair always obtained the larger reward whenever they agreed to pull the unequal tray. The continuous lines describe the predicted outcome for the case in which individuals in a pair alternated who obtained the larger reward whenever they cooperated in order to obtain the unequal share. The bigger black dots represent five (three or two) data points. N = 12 for each age group.

The Monte Carlo simulations revealed that at both ages children did not choose the apparatus (equal vs. unequal) randomly, and exhibited a preference for one or the other apparatus (3.5 and 5-year-olds: p = 0.0001). The second Monte Carlo simulation revealed that at both ages children chose the unequal apparatus significantly more often than expected by chance (3.5 and 5-year-olds: p = 0.0001, Fig. 5a and 5b).

As in Experiment 1 we investigated whether or not there was an overall fair distribution of rewards when children chose the unequal apparatus (due to alternation regarding who obtained the large amount). The final distribution of gummy bears did not differ significantly from chance levels in either age group (Wilcoxon signed-rank test, 3.5-year-olds: T = 46, N = 11, p = 0.25; 5-year-olds: T = 40, N = 10, p = 0.21). This is because there were 5 and 4 dyads of the 3.5-year and 5-year-olds respectively, in which there was some alternation between individuals regarding who obtained the large amount.

The more unequal outcome of this experiment was also revealed in the analysis that looked at the first choices made. We found that at both ages, at trial 1, children chose the equal apparatus less often than expected by chance (only 1 out of 12 dyads in both age groups chose the equal apparatus, binomial test: p = 0.006). If we look at the data and split the results based on gender and familiarity of the partner, there is also no difference. Both boys and girls and familiar partners and non-familiar partners chose the equal apparatus significantly less often than expected by chance (boys: p<0.001; girls: p = 0.038; familiar partners: p = 0.006; non-familiar partners: p = 0.006).

The characterization of the dyads’ general pattern as “inequality”, “equality”, “reciprocal” and “mixed” revealed that eight dyads of 3.5-year-olds exhibited mostly inequality, one dyad chose mostly “equal” and three reciprocally. Among the 5-year-olds, nine out of the 12 dyads exhibited inequality, one equality and two behaved reciprocally (Fig. 2b).

#### Description of the negotiation process

First, we looked at children’s protests at a descriptive level and found that on average, 3.5-year-olds protested in 52% of trials (only 1 out of 12 dyads never protested) and 5-year-olds in 42% of trials (again, only 1 dyad never protested). In most of the cases these protests were against pulling for the small amount (92% and 95% of protests among the 3.5-year-olds and 5-year-olds respectively). A small proportion of the protests were also against pulling the equal tray (8% and 5% of protests among the 3.5-year-olds and 5-year-olds respectively), which often occurred after the partner had obtained the large amount from the unequal apparatus in previous trials.

In this experiment we couldn’t analyse the negotiation process as in Experiment 1 (comparing the children’s behaviour prior to pulling the equal or the unequal tray) since only one dyad at each age group agreed on pulling the equal tray. Children accepted the unequal tray immediately in 42% and 40% of the trials (3.5- and 5-year-olds respectively). In the remaining 58% and 60% (3.5- and 5-year-olds respectively) of the trials that ended in an unequal outcome there was some communication or discussion between partners.

### Discussion

Dyads at both ages agreed in the majority of the trials. However, levels of agreement were slightly lower than in the previous experiment, in particular among the 5-year-olds. Three and 5-year-olds agreed almost exclusively on the unequal split, and they did this from trial 1. When looking at the distribution of rewards after pulling the unequal apparatus, we found that both age groups did not differ from chance, meaning that they were not completely unfair (one child monopolizing always the large amount) but also not completely fair (taking turns). This is because, despite the majority of dyads agreeing on a rather unequal deal, a small number of dyads at both ages took turns obtaining the large amount of the unequal tray. Thus, the higher inequality of this experiment did not lead to pure equal outcomes among the 3.5 or 5 year-olds. Children in the advantageous position neither suggested pulling for the equal split, nor did children in the disadvantageous position manipulate their partners into pulling the equal tray by refusing to pull. Basically, we can conclude that the behaviour of the 3.5-year-olds was similar to that of the previous experiment, whereas 5-year-olds exhibited more inequality. Very few dyads in both age groups (3 per age group) developed an alternating fair strategy.

We found a gender and familiarity effect, with boys and familiar partners sharing less fairly than girls and unfamiliar partners respectively. The gender effect in this experiment would be in line with the results from several developmental studies that have reported that girls behave more pro-socially than boys [5, 16, 28, 32, 33]. However, many other studies have not found gender difference or have even found differences in the other direction [1]. Familiar partners in this experiment agreed on an unequal distribution of rewards more often than unfamiliar partners. This contrasts with the results from sharing and pro-social studies in which friendship positively influences the allocation of resources and pro-social interventions [10, 34, 35]. However, we did not have proper measures on friendship, and therefore although we can be sure that our familiar partners knew each other and did not dislike each other, we cannot be sure that they were really good friends. Furthermore, a bargaining study [21] reports similar findings among 10 and 12-years-old friends. Although friends prefer equal distributions, they will accept inequality if the partner really wants the reward. As suggested by Morgan & Sawyer [21], it is possible that friends are more willing to tolerate unequal distributions than non-friends are, and that they do not care that much about temporary imbalances between them since in the long run it is very likely that things will balance out.

## General Discussion

The results of these two experiments show that children of 3.5 and 5 years of age do not choose an equal allocation of rewards in a face-to-face bargaining situation. This seems to be the case even when children depend on each other and have the option of communicating and negotiating with the partner by way of face-to-face interaction. In Experiment 1, 3.5-year-olds agreed mostly on an unequal outcome, whereas 5-year-olds exhibited less inequality than 3.5-year-olds but still did not preferentially agree on the equal outcome. In Experiment 2 children at both ages exhibited mostly inequality and some few pairs developed a turn-taking and fair strategy.

The inequality observed in Experiment 2 among the 5-year-olds (who in Experiment 1 exhibited less inequality than the 3.5-year-olds) could be due to the higher costs involved at giving up the unequal tray in this last experiment. In Experiment 1 the cost to keep the partner happy was smaller (1 reward) than in Experiment 2, where one child had to forego 4 rewards to maintain equality. Several other studies have also found that equality is often influenced by the costs subjects must endure to maintain it [7, 12].

The results of this study show that in conflict-of-interest situations in which 3.5 and 5-year-olds have a choice between an unequal and an equal split, the outcome is mostly unequal. Children in the most favourable position were willing and happy to obtain the large amount, independently of the consequences that this had for their partner, showing no aversion to advantageous inequality [12, 14, 36]. Children in the disadvantageous position talked and often expressed their disagreement and unhappiness about obtaining the small amount, which also supports previous findings of disadvantageous inequality aversion at these ages [14, 15]. However, they were mostly unable to suggest and persuade their partner to agree on an equal and egalitarian distribution of the rewards, so that overall inequality prevailed. Although the overall pattern was a rather unequal allocation of rewards, we cannot rule out the possibility that the outcome would have been even more unequal had children been allowed to make unilateral decisions, without having the opportunity to negotiate

In any case, the results are particularly surprising considering the fact that children were required to collaborate by way of face-to-face interaction with peers from the same school and to do so repeatedly, which is supposed to influence pro-sociality positively. Whenever there is a possibility for future interaction and in particular if subjects are dependent on each other (as they were in this task, in which they needed the partner to succeed), subjects might behave pro-socially because of the expectation of future benefits (or future help needed) from the partner. Of course, this argument could be an explanation for children’s acceptance to pull the unequal and disadvantageous split. Actually, there were trials in which children convinced their partners to leave the equal tray and pull the unequal tray, or change positions in the unequal tray to their advantage. This interpretation would also be supported by the findings that outcomes among familiar partners were more unequal than among unfamiliar partners. However, it was never the disadvantaged child’s initiative, and we cannot know whether children were motivated by friendship considerations or were simply trying to avoid conflict. Since children never agreed or promised each other to share the resources after the test or outside the test room, we can also rule out the hypothesis that children accepted the disadvantageous splits expecting future sharing from the partner.

Furthermore, there were also those trials in which children expressed their unhappiness about obtaining just one gummy bear, physically competing over the position in front of the large amount, or verbally expressing that they also wanted a large amount and did not want the small amount. Surprisingly, in these cases children very rarely suggested to pull the equal tray. Only among the 5-years-olds did one girl suggested to pull the equal tray saying that “that was the fair thing to do”. So, despite children protesting and communicating in various ways their preference for a larger reward [15], their partners were not particularly reactive to their desires, and were not willing to compromise and pay a cost in favour of a more egalitarian distribution of rewards, either by choosing the equal tray or by taking turns at obtaining the largest amount. Although Brownell et al. [9] found that 24-month-olds chose the equal option more often when the recipient communicated her interest in the rewards, their results do not conflict with ours, since in their study choosing the equal outcome was not associated with higher costs (children benefited equally sharing or not sharing with the partner). Therefore, our current results do not support the hypothesis that young children’s low sharing levels is due to the difficulty in recognizing others’ needs or desires [23]. Although this is a possible explanation for children’s prosocial behaviour in other contexts and at younger ages [9], in the current interactive situation children clearly knew their partner’s preference.

So, why can’t children agree on pulling the fair split in this task? First, although children were required to pull together, the decision whether to acquire the unequal or the equal distribution of rewards was made prior to collaboration, resembling more a windfall situation, in which individuals are confronted with non-collaboratively produced resources. This would support previous findings and theories suggesting a different developmental trajectory for sharing resources acquired in different ways [37]. In studies in which rewards are produced collaboratively, 3.5-year-olds share equally [17–19], whereas in the context of non-collaboratively produced resources it is only after 5 years of age when children become averse to advantageous inequality and start incorporating fairness norms in their interactions with others [7, 10–12,14,16]. Although 3.5-year-olds are sensitive to disadvantageous inequality and are willing to pay a cost to reject bad deals, their behaviour is biased towards self-interest. Several studies have also shown that although 3.5-year-olds reject disadvantageous allocations, levels of rejection still increase with age. Furthermore, 3.5- to 5-year-olds will not forego benefits to correct advantageous inequality or obtain less than a partner who deserves more at these young ages [12,14, 16,38–41].

There are still a number of aspects that deserve further investigation to fully understand what promotes and limits equality at these young ages. First, would the results be any different if they first worked together obtaining the resources and then had to make a choice between equal and unequal allocations? If this was the case, this would further support the collaboration explanation. If not, it would suggest that there are additional variables influencing how children share at these ages, and joint acquisition of resources does not automatically guarantee equality.

It is also possible that the present task is cognitively more complicated than actively dividing a pile of clumped resources after collaboration or restoring equality by giving up one reward after a 3–1 split [17,18]. In this task with predetermined allocations, that were in addition spatially separated, children had to keep in mind the two alternative options (which included three different quantities) and the difference in benefits not only in relationship to the alternative tray but also in relationship to each other. Therefore, a similar study in which children can see at once both alternative trays (maybe sitting across each other over a table) would clarify whether part of the difficulty of the present task was that children could not keep in mind all alternative options and how they compared to each other. Furthermore, since the two predetermined distributions of rewards were presented to the children as two equally possible outcomes by the adult experimenters, it is possible that children might have considered it warranted by the adults to aim for the largest amount of rewards even when their partner would obtain a smaller split. This would suggest that the adult-defined social/normative context strongly influences whether or not children allocate resources in an egalitarian way at these young ages.

In the future, we will also need dominance data that allow us to test whether children obtaining the large amount were also more dominant ones. Some previous studies measuring dominance have shown that dominance does indeed predict levels of resource control and utilization at these young ages [42–44]. Dominant children used both prosocial and coercive strategies to monopolize the primary (and better) role in a play interaction with a peer [44]. Alternatively, it could be that it is not dominance per se but rather other skills (e.g. levels of inhibitory control) what allows children to dominate resources in such a task. Interestingly, a similar study with chimpanzees, who lack fairness norms and do not show inequity aversion [45], showed that some dyads end up choosing equal outcomes because individuals are capable of outwaiting their despotic partners who finally accept the equal option [46].

To sum up, the present study shows that in face-to-face bargaining situations in which children have to choose between an equal or an unequal distribution of non-collaboratively produced rewards they choose unequal allocations. Despite interdependence between partners and the possibility to protest, communicate, and refuse to pull, equality does not emerge at these young ages.

## Supporting Information

S1 Fig Proportion of trials which outcome was immediately accepted without any discussion or negotiation and proportion of trials which outcome was due to a change of tray in Experiment 1. (TIF)

S1 Text Supplemental text. (DOCX)
