# Supplementary figures and images for: Non-Egalitarian Allocations among Preschool Peers in a Face-to-Face Bargaining Task
Source: PLoS One. 2015 Mar 18;10(3):e0120494. doi: 10.1371/journal.pone.0120494 (PMC4364954; doi:10.1371/journal.pone.0120494)

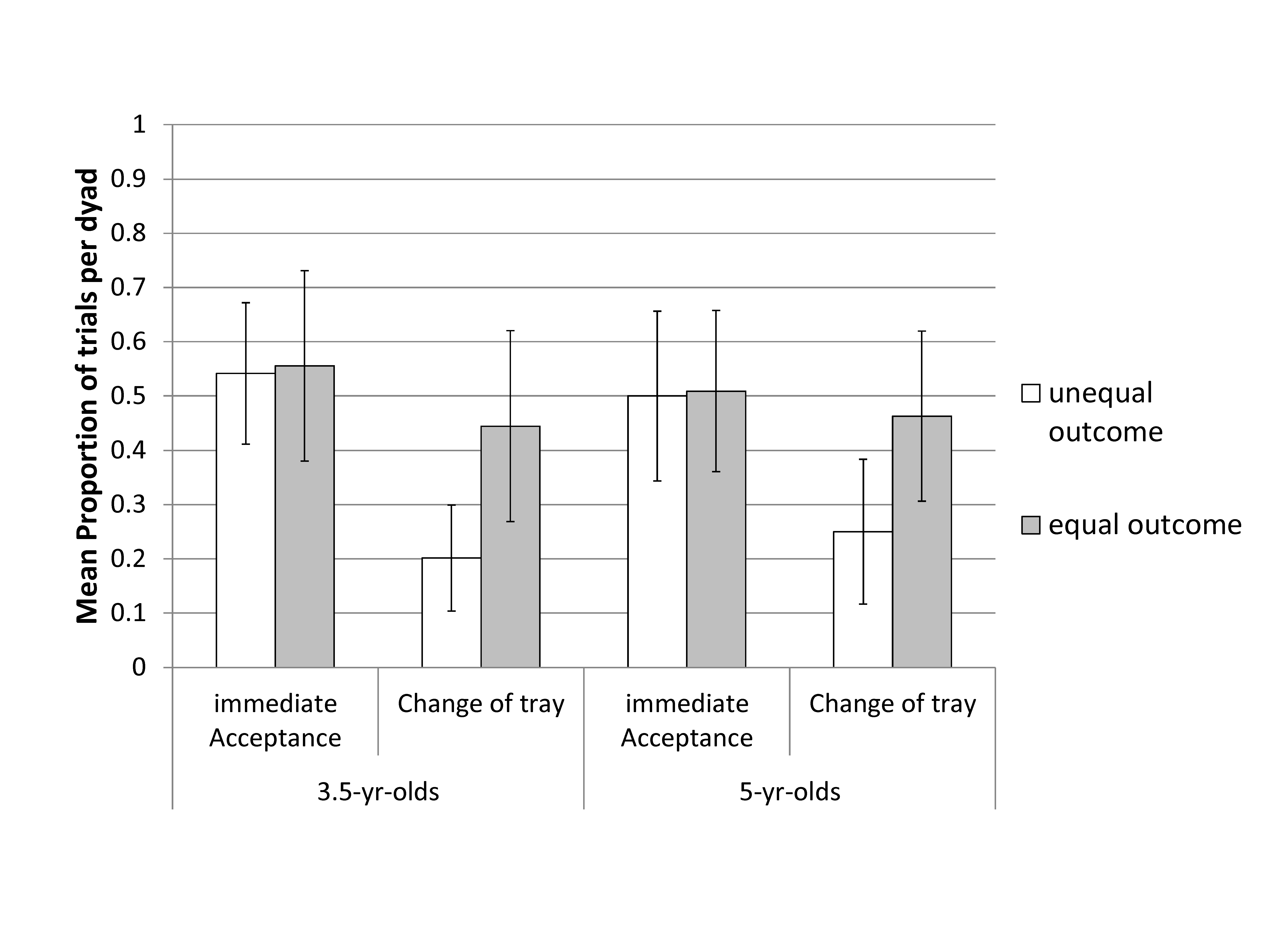

Supplement: S1 Fig — (TIF) [file pone.0120494.s001.tif]
