# Supplementary material for: Non-Egalitarian Allocations among Preschool Peers in a Face-to-Face Bargaining Task
Source: PLoS One. 2015 Mar 18;10(3):e0120494. doi: 10.1371/journal.pone.0120494 (PMC4364954; doi:10.1371/journal.pone.0120494)
Supplement: S1 Text — (DOCX) [file pone.0120494.s002.docx]

**Supporting Information**

**Experiment 1**

The GLMs were conducted as follows. Since the response was a count in each case we used a model with Poisson family and log link function. For none of these models was overdispersion (Agresti, 1996) indicated to be an issue (all p > 0.999). To account for the differences in the number of valid trials per dyad, we included the natural log of this number as an offset variable in the models. For the response measure “trials where the unequal apparatus was chosen”, the offset variable was the number of trials with agreement (per dyad), and for the difference of gummy bears within the dyads the offset variable was the total amount of gummy bears obtained per dyad. In a first step we tested the full model against the null model using a likelihood ratio test (Dobson, 2002). Only if this revealed significance did we then check for the significance of the predictor variables. The second step was the removal of non-significant interactions (p > .20) in a stepwise manner. The significance of the remaining variables and interactions was then obtained from this reduced model.

We calculated the distribution of the gummy bears as follows: E= payoff of the equal split, S= payoff of the small reward for the unequal split, L= payoff of the large reward for the unequal split. We considered: E= 0.5 (two of four possible gummy bears), S= 0.25 (one of four) and L= 0.75 (three of four). The total payoff obtained by the highest earner of each dyad was calculated in the following way: B= a * 0.75 + b * 0.25 + c * 0.5. Here, a = number of trials in which they pulled the unequal apparatus and child obtained the large amount, b= number of trials in which they pulled the unequal apparatus and child obtained the small amount and c= number of trials in which they pulled the equal apparatus.

The maximal potential benefit for a child (B_max_) who never allows the partner to obtain the larger amount of gummy bears when they cooperate to obtain the unequal split was calculated as follows:

If x = proportion of cooperation for unequal apparatus and 1-x = proportion of cooperation for equal apparatus,

B_max_ = (x * L) + (1-x) * E

If L = 0.75 and E = 0.5, we obtain: B_max_ = 0.25 * x + 0.5

If we include the option of alternation across unequal trials (with both children per dyad obtaining the large reward as many times as they do the small reward), we obtain:

B_max_ = (x * L)/2 + (x * S)/2 + (1-x) * E

If L = 0.75 and E = 0.5, we obtain: B_max_ = 0.5

Note that these are two approximate functions, since an odd number of unequal trials does not allow for an exact split of rewards.

**Additional results**

Table S2A: Predictors influencing the difference of gummy bears within dyads in Experiment1.

| Predictor variable | Estimate | SE | Z | P |
| --- | --- | --- | --- | --- |
| Intercept | -1.649 | 0.239 |  |  |
| Gender | 0.454 | 0.246 | 1.843 | 0.065 |
| Age | -0.519 | 0.246 | -2.112 | 0.035 |
| Familiarity of partner | 0.133 | 0.237 | 0.562 | 0.574 |

**Experiment 2**

In this experiment, as in the previous one, E = payoff of the equal split, S= payoff of the small reward for the unequal split, L= payoff of the large reward for the unequal split. Since E= 0.29 (two out of seven), S= 0.14 (one out of seven) and L= 0.86 (six out of seven). Therefore, the total payoff obtained by the highest earner of each dyad was calculated in the following way: B= a * 0.86 + b * 0.14 + c * 0.29.

If x = proportion of cooperation for unequal apparatus and 1-x = proportion of cooperation for equal apparatus, the maximal potential benefit for a child (B_max_), who never allows the partner to obtain the larger amount of gummy bears when they cooperate to obtain the unequal split is:

B_max_ = (x * L) + (1-x) * E

If L = 0.86 and E = 0.29, we obtain: B_max_ = 0.57 * x + 0.29

If we include the option of alternation across unequal trials (with both children per dyad obtaining the large reward as many times as they do the small reward), we obtain:

B_max_ = (x * L)/2 + (x * S)/2 + (1-x) * E

If L = 0.86 and E = 0.29, we obtain: B_max_ = 0.21 * x + 0.29

**Additional results**

Table S2B: Predictors influencing the difference of gummy bears within dyads in Experiment 2.

|  | Estimate | SE | Z | p |
| --- | --- | --- | --- | --- |
| Intercept | -0.96 | 0.1457 |  |  |
| Gender | -0.318 | 0.138 | -2.311 | 0.020 |
| Age | 0.064 | 0.137 | 0.467 | 0.640 |
| Familiarity of partner | 0.391 | 0.140 | 2.790 | 0.005 |

**References**

Agresti, A. (1996). An introduction to categorical data analysis. John Wiley and Sons, New York, NY.

Dobson, A. J. (2002). An introduction to generalized linear models. Chapman and Hall, London.
